# Supplementary material for: Modeling glioblastoma heterogeneity as a dynamic network of cell states
Source: Mol Syst Biol. 2021 Sep 16;17(9):e10105. doi: 10.15252/msb.202010105 (PMC8444284; doi:10.15252/msb.202010105)
Supplement: Supplementary file 6 — Source Data for Figure 5 [file MSB-17-e10105-s004.zip › Figure5A_sourcedata/GSEA_3071/hallmarks_stateVI.GseaPreranked.1623742557031/HALLMARK_G2M_CHECKPOINT.html]

Details for gene set HALLMARK\_G2M\_CHECKPOINT[GSEA]

|  || Dataset | state63071 |
| Phenotype | NoPhenotypeAvailable |
| Upregulated in class | na\_neg |
| GeneSet | HALLMARK\_G2M\_CHECKPOINT |
| Enrichment Score (ES) | -0.2890121 |
| Normalized Enrichment Score (NES) | -1.4029533 |
| Nominal p-value | 0.06964091 |
| FDR q-value | 0.30238566 |
| FWER p-Value | 0.985 |
Table: GSEA Results Summary

  

Fig 1: Enrichment plot: HALLMARK\_G2M\_CHECKPOINT      
 Profile of the Running ES Score & Positions of GeneSet Members on the Rank Ordered List

  

| PROBE | GENE SYMBOL | GENE\_TITLE | RANK IN GENE LIST | RANK METRIC SCORE | RUNNING ES | CORE ENRICHMENT || 1 | HMGA1 |  |  | 1 | 1.500 | 0.0325 | No |
| 2 | CDC20 |  |  | 15 | 1.073 | 0.0523 | No |
| 3 | ODC1 |  |  | 38 | 0.918 | 0.0662 | No |
| 4 | MT2A |  |  | 58 | 0.859 | 0.0796 | No |
| 5 | MYC |  |  | 69 | 0.815 | 0.0946 | No |
| 6 | SLC38A1 |  |  | 178 | 0.622 | 0.0778 | No |
| 7 | PTTG1 |  |  | 344 | 0.514 | 0.0425 | No |
| 8 | PRMT5 |  |  | 372 | 0.502 | 0.0459 | No |
| 9 | ATF5 |  |  | 401 | 0.490 | 0.0487 | No |
| 10 | DKC1 |  |  | 430 | 0.478 | 0.0512 | No |
| 11 | TROAP |  |  | 479 | 0.461 | 0.0478 | No |
| 12 | AMD1 |  |  | 537 | 0.444 | 0.0414 | No |
| 13 | AURKA |  |  | 572 | 0.432 | 0.0413 | No |
| 14 | HMMR |  |  | 681 | 0.404 | 0.0197 | No |
| 15 | PML |  |  | 874 | 0.365 | -0.0264 | No |
| 16 | DDX39A |  |  | 936 | 0.355 | -0.0358 | No |
| 17 | JPT1 |  |  | 965 | 0.350 | -0.0361 | No |
| 18 | CCNB2 |  |  | 967 | 0.350 | -0.0287 | No |
| 19 | UBE2S |  |  | 974 | 0.349 | -0.0228 | No |
| 20 | TOP1 |  |  | 975 | 0.349 | -0.0151 | No |
| 21 | SLC7A5 |  |  | 989 | 0.348 | -0.0112 | No |
| 22 | CDC27 |  |  | 1259 | 0.308 | -0.0802 | No |
| 23 | NEK2 |  |  | 1307 | 0.302 | -0.0869 | No |
| 24 | HIF1A |  |  | 1458 | 0.284 | -0.1229 | No |
| 25 | KPNB1 |  |  | 1538 | 0.275 | -0.1392 | No |
| 26 | RAD23B |  |  | 1586 | 0.270 | -0.1465 | No |
| 27 | ORC5 |  |  | 1594 | 0.269 | -0.1426 | No |
| 28 | PLK1 |  |  | 1665 | 0.263 | -0.1566 | No |
| 29 | BIRC5 |  |  | 1667 | 0.263 | -0.1511 | No |
| 30 | NOLC1 |  |  | 1699 | 0.260 | -0.1542 | No |
| 31 | ILF3 |  |  | 1806 | -0.250 | -0.1786 | No |
| 32 | MKI67 |  |  | 1825 | -0.253 | -0.1781 | No |
| 33 | HMGN2 |  |  | 1857 | -0.258 | -0.1812 | No |
| 34 | STAG1 |  |  | 1895 | -0.263 | -0.1859 | No |
| 35 | AURKB |  |  | 1978 | -0.274 | -0.2030 | No |
| 36 | KIF22 |  |  | 2014 | -0.281 | -0.2067 | No |
| 37 | PRPF4B |  |  | 2062 | -0.288 | -0.2136 | No |
| 38 | KIF11 |  |  | 2121 | -0.301 | -0.2234 | No |
| 39 | TFDP1 |  |  | 2143 | -0.304 | -0.2227 | No |
| 40 | POLE |  |  | 2154 | -0.306 | -0.2188 | No |
| 41 | POLQ |  |  | 2169 | -0.308 | -0.2160 | No |
| 42 | CDKN2C |  |  | 2197 | -0.313 | -0.2168 | No |
| 43 | RPS6KA5 |  |  | 2215 | -0.317 | -0.2146 | No |
| 44 | ORC6 |  |  | 2343 | -0.342 | -0.2430 | No |
| 45 | CCNF |  |  | 2350 | -0.343 | -0.2371 | No |
| 46 | STIL |  |  | 2376 | -0.349 | -0.2366 | No |
| 47 | PBK |  |  | 2412 | -0.356 | -0.2387 | No |
| 48 | SAP30 |  |  | 2413 | -0.356 | -0.2309 | No |
| 49 | MCM5 |  |  | 2424 | -0.359 | -0.2258 | No |
| 50 | EXO1 |  |  | 2516 | -0.381 | -0.2431 | No |
| 51 | SMARCC1 |  |  | 2522 | -0.383 | -0.2362 | No |
| 52 | SMC4 |  |  | 2634 | -0.408 | -0.2585 | No |
| 53 | STMN1 |  |  | 2646 | -0.411 | -0.2527 | No |
| 54 | CDC45 |  |  | 2776 | -0.448 | -0.2792 | Yes |
| 55 | RAD21 |  |  | 2797 | -0.457 | -0.2749 | Yes |
| 56 | CTCF |  |  | 2820 | -0.462 | -0.2710 | Yes |
| 57 | MCM6 |  |  | 2834 | -0.468 | -0.2644 | Yes |
| 58 | CENPF |  |  | 2842 | -0.472 | -0.2561 | Yes |
| 59 | KIF15 |  |  | 2855 | -0.478 | -0.2490 | Yes |
| 60 | SMC2 |  |  | 2864 | -0.483 | -0.2407 | Yes |
| 61 | ARID4A |  |  | 2907 | -0.497 | -0.2417 | Yes |
| 62 | BRCA2 |  |  | 2916 | -0.500 | -0.2330 | Yes |
| 63 | CDC6 |  |  | 2990 | -0.534 | -0.2419 | Yes |
| 64 | NSD2 |  |  | 3002 | -0.544 | -0.2331 | Yes |
| 65 | KNL1 |  |  | 3019 | -0.550 | -0.2256 | Yes |
| 66 | TMPO |  |  | 3022 | -0.552 | -0.2141 | Yes |
| 67 | CHAF1A |  |  | 3023 | -0.552 | -0.2020 | Yes |
| 68 | ATRX |  |  | 3036 | -0.557 | -0.1932 | Yes |
| 69 | NASP |  |  | 3049 | -0.563 | -0.1843 | Yes |
| 70 | RBL1 |  |  | 3063 | -0.569 | -0.1755 | Yes |
| 71 | MCM3 |  |  | 3064 | -0.570 | -0.1630 | Yes |
| 72 | SMC1A |  |  | 3067 | -0.571 | -0.1511 | Yes |
| 73 | LBR |  |  | 3068 | -0.572 | -0.1386 | Yes |
| 74 | FOXN3 |  |  | 3103 | -0.587 | -0.1354 | Yes |
| 75 | CASP8AP2 |  |  | 3111 | -0.593 | -0.1244 | Yes |
| 76 | GINS2 |  |  | 3119 | -0.598 | -0.1133 | Yes |
| 77 | MEIS1 |  |  | 3133 | -0.610 | -0.1036 | Yes |
| 78 | NUMA1 |  |  | 3136 | -0.611 | -0.0908 | Yes |
| 79 | E2F1 |  |  | 3140 | -0.613 | -0.0783 | Yes |
| 80 | PDS5B |  |  | 3177 | -0.641 | -0.0744 | Yes |
| 81 | MYBL2 |  |  | 3196 | -0.660 | -0.0651 | Yes |
| 82 | NUSAP1 |  |  | 3199 | -0.663 | -0.0511 | Yes |
| 83 | LMNB1 |  |  | 3246 | -0.711 | -0.0486 | Yes |
| 84 | BARD1 |  |  | 3277 | -0.736 | -0.0409 | Yes |
| 85 | EZH2 |  |  | 3282 | -0.741 | -0.0259 | Yes |
| 86 | CDK1 |  |  | 3396 | -0.883 | -0.0384 | Yes |
| 87 | FBXO5 |  |  | 3413 | -0.919 | -0.0228 | Yes |
| 88 | CDKN1B |  |  | 3426 | -0.951 | -0.0054 | Yes |
| 89 | TOP2A |  |  | 3457 | -1.020 | 0.0084 | Yes |
| 90 | MEIS2 |  |  | 3611 | -1.934 | 0.0076 | Yes |
Table: GSEA details [plain text format]

  

Fig 2: HALLMARK\_G2M\_CHECKPOINT: Random ES distribution      
 Gene set null distribution of ES for **HALLMARK\_G2M\_CHECKPOINT**
